# Complete Genome Sequences of Two Interactive Moderate Thermophiles, *Paenibacillus napthalenovorans* 32O-Y and *Paenibacillus* sp. 32O-W

**DOI:** 10.1128/genomeA.01717-15

**Published:** 2016-02-11

**Authors:** Robert R. Butler, Jia Wang, Benjamin C. Stark, Jean-François Pombert

**Affiliations:** Department of Biology, Illinois Institute of Technology, Chicago, Illinois, USA

## Abstract

Microorganisms with the capability to desulfurize petroleum are in high demand with escalating restrictions currently placed on fuel purity. Thermophilic desulfurizers are particularly valuable in high-temperature industrial applications. We report the whole-genome sequences of *Paenibacillus napthalenovorans* 32O-Y and *Paenibacillus* sp. 32O-W, which can and cannot, respectively, metabolize dibenzothiophene.

## GENOME ANNOUNCEMENT

Biodesulfurization of petroleum is critical in the face of escalating restrictions on fuel purity, and alkylated derivatives of dibenzothiophene (DBT) are among the most common contaminants in petroleum fuel precursors (1). Thermophilic microorganisms capable of desulfurization are especially valuable, as they can be used in high temperature industrial settings (2–5). Previous research isolated two bacterial strains, *Paenibacillus napthalenovorans* 32O-Y, which can metabolize DBT, and *Paenibacillus* sp. 32O-W, which cannot. Curiously, when the two strains are in a mixed culture, 32O-Y metabolizes DBT at a higher rate than in isolation (6). Here, we present the whole-genome sequences of both 32O-Y and 32O-W.

Isolation, purification, and culture of the two species were previously described (6). Genomic DNA was extracted using the PowerSoil (MoBio, Carlsbad, CA, USA) and MasterPure Gram-positive (Epicentre, Madison, WI, USA) DNA purification kits. Long-read sequencing was conducted on a PacBio RS II sequencer (Pacific Biosciences, Menlo Park, CA, USA) using two single-molecule real-time cells and P4-C2 chemistry at the University of Michigan DNA Sequencing Core (Ann Arbor, MI, USA). DNA was purified using AMPure XP beads (Beckman Coulter, Brea, CA, USA) and sheared to 10 kbp (*N*_50_s: 32O-W, 6,562 bp; 32O-Y, 7,096 bp) with g-TUBEs (Covaris, Woburn, MA, USA). Additional short-read sequencing was conducted on a MiSeq sequencer (Illumina, San Diego, CA, USA) at Université Laval’s Plate-forme d’Analyses Génomiques (Québec, QC, Canada). A 300-bp paired-end protocol was run on TruSeq (Illumina) DNA libraries (insert sizes: 32O-W, 392 ± 106 bp; 32O-Y, 433 ± 123 bp).

Assembly of 32O-W (5,200,139 bp, 56.34% G+C, 93× coverage) was completed using the HGAP2 protocol in SMRT-Analysis version 2.2.0 (7). The assembly was trimmed and then corrected via sequential runs of the RS_Resequencing.1 protocol. Base-calling was further polished by mapping Illumina reads (169× coverage) to the assembly using Geneious version 7.1.9 (8). HGAP2 assembly of 32O-Y produced six contigs. Illumina reads were assembled *de novo* using SPAdes version 3.5.0 (9) and Geneious and compared to the HGAP2 assembly. 32O-Y HGAP2 contigs were joined using overlaps from the other assemblies. This assembly (5,375,858 bp, 49.69% G+C; PacBio 109×, Illumina 122×) was then trimmed and corrected as above.

Genomes were annotated using Prokka version 1.11 (10), with a database containing six reference genomes (*Paenibacillus curdlanolyticus* YK9, NZ_AEDD01000040; *Paenibacillus darwinianus*, NZ_KK082115; *Paenibacillus harenae* DSM 16969, NZ_KE383842; *Paenibacillus pinihumi* DSM 23905, NZ_KE383864; *Paenibacillus* sp. G1, NZ_CBVJ010000001; *Paenibacillus* sp. JDR-2, NC_012914). The RNAmmer version 1.2 (11) switch was used for rRNA annotation, and the tRNA annotations were verified with tRNAscan-SE version 1.3.1 (12). Hypothetical protein annotations were checked with InterProScan versions 5.14-53.0 (13), and conserved domain hits above 1 × 10^−10^ were amended. An NCBI genome submission check (https://www.ncbi.nlm.nih.gov/genomes/frameshifts/frameshifts.cgi) identified potential pseudogenes (three in 32O-W, seven in 32O-Y). A total of 5,054/4,752 coding sequences, 94/69 tRNAs, 34/19 rRNAs, 1/7 repeat regions, 1/1 tmRNA, and 382/439 signal peptides were annotated in the 32O-Y/32O-W genomes.

### Nucleotide sequence accession numbers.

Whole-genome sequences were deposited in GenBank under the accession numbers CP013652 (32O-Y) and CP013653 (32O-W). Both species are in the ARS Culture Collection, Peoria, IL, USA, under NRRL catalog numbers B-65351 and B-65352.
